# Disease-specific monoclonal antibodies targeting glutamate decarboxylase impair GABAergic neurotransmission and affect motor learning and behavioral functions

**DOI:** 10.3389/fnbeh.2015.00078

**Published:** 2015-03-27

**Authors:** Mario Manto, Jérôme Honnorat, Christiane S. Hampe, Rafael Guerra-Narbona, Juan Carlos López-Ramos, José María Delgado-García, Fumihito Saitow, Hidenori Suzuki, Yuchio Yanagawa, Hidehiro Mizusawa, Hiroshi Mitoma

**Affiliations:** ^1^Unité d’Etude du Mouvement, FNRS Neurologie, ULB ErasmeBrussels, Belgium; ^2^Neuroscience Research Center INSERMLyon, France; ^3^School of Medicine, University of WashingtonSeattle, WA, USA; ^4^Division of Neurosciences, Pablo de Olavide UniversitySeville, Spain; ^5^Department of Pharmacology, Nippon Medical SchoolTokyo, Japan; ^6^Department of Genetic and Behavioral Neuroscience, Gunma University Graduate School of Medicine and JST, CREST, Maebashi CityGunma, Japan; ^7^National Center of Neurology and PsychiatryTokyo, Japan; ^8^Department of Medical Education, Tokyo Medical UniversityTokyo, Japan

**Keywords:** autoantibodies, GAD65, GABAergic neurotransmission, stiff person syndrome, epitopes

## Abstract

Autoantibodies to the smaller isoform of glutamate decarboxylase (GAD) can be found in patients with type 1 diabetes and a number of neurological disorders, including stiff-person syndrome, cerebellar ataxia and limbic encephalitis. The detection of disease-specific autoantibody epitopes led to the hypothesis that distinct GAD autoantibodies may elicit specific neurological phenotypes. We explored the *in vitro/in vivo* effects of well-characterized monoclonal GAD antibodies. We found that GAD autoantibodies present in patients with stiff person syndrome (*n* = 7) and cerebellar ataxia (*n* = 15) recognized an epitope distinct from that recognized by GAD autoantibodies present in patients with type 1 diabetes mellitus (*n* = 10) or limbic encephalitis (*n* = 4). We demonstrated that the administration of a monoclonal GAD antibody representing this epitope specificity; (1) disrupted *in vitro* the association of GAD with γ-Aminobutyric acid containing synaptic vesicles; (2) depressed the inhibitory synaptic transmission in cerebellar slices with a gradual time course and a lasting suppressive effect; (3) significantly decreased conditioned eyelid responses evoked in mice, with no modification of learning curves in the classical eyeblink-conditioning task; (4) markedly impaired the facilitatory effect exerted by the premotor cortex over the motor cortex in a paired-pulse stimulation paradigm; and (5) induced decreased exploratory behavior and impaired locomotor function in rats. These findings support the specific targeting of GAD by its autoantibodies in the pathogenesis of stiff-person syndrome and cerebellar ataxia. Therapies of these disorders based on selective removal of such GAD antibodies could be envisioned.

## Introduction

Conversion of glutamate to GABA is catalyzed by two isoforms of glutamate decarboxylase (GAD): GAD67 and GAD65. Autoantibodies to the smaller isoform (GAD65Ab) can be found in patients with type 1 diabetes, and a number of neurological disorders, including stiff-person syndrome (SPS), cerebellar ataxia (Björk et al., 1994; Honnorat et al., 2001; Rakocevic et al., 2006), and limbic encephalitis (Matà et al., 2010) while GAD67 is rarely autoantigenic.

The hypothesis that GAD65Ab specifically target and impair GAD65 function in neurological diseases is supported by passive transfer experiments of GAD65Ab. *In vivo* injections of rat or mouse brain with monoclonal GAD65Ab or purified immunoglobulin obtained from GAD65Ab-positive sera of SPS patients induced increased excitability of the spinal cord (Manto et al., 2011), increase of neuronal synaptic function (Vega-Flores et al., 2014), stiffness-like motor deficits (Hansen et al., 2013), behavioral changes including anxiety (Geis et al., 2011), and changes in cognitive functions (Hampe et al., 2013).

In our previous studies we established that monoclonal GAD65Ab with different epitope specificities induced distinct neurological changes when injected *in vivo*. This strongly supports our hypothesis that distinct neurological symptoms in GAD65Ab-associated neurological diseases and the apparent lack of such symptoms in patients with type 1 diabetes are caused by disease-specific GAD65Ab (Manto et al., 2007, 2011; Hampe et al., 2013; Vega-Flores et al., 2014). However, it remained crucial to establish GAD65 as the pathogenic target of GAD65Ab.

We performed novel studies towards the pathogenic mechanisms involved, demonstrating that patients with SPS and cerebellar ataxia develop GAD65Ab recognizing disease-specific epitopes, and inhibiting the enzyme activity of GAD65. We analyzed the effect of GAD65-specific monoclonal antibodies on the co-localization of GAD65 with GABAergic synaptic vesicles and cerebellar GABAergic inhibition in rats as well as in wild-type (WT) and GAD65-knockout (KO) mice. Subsequently we relayed our findings to the interpretation of *in vivo* studies of the effects of GAD65-specific monoclonal antibodies on (a) learning and memory acquisition in mice (classical eyeblink conditioning); (b) corticomotor responses in rats; and (c) anxiety-related behavior in rats.

## Materials and Methods

### Patients

Sera of patients diagnosed with cerebellar ataxia (*n* = 15), SPS (*n* = 7), and limbic encephalitis (*n* = 4) were included in this study. Ten patients diagnosed with type 1 diabetes without neurological symptoms were included as controls. Clinical parameters including age, gender, neurological diagnosis, and presence of other autoimmune diseases are summarized in Table 1 along with GAD65Ab results, including titer and epitope specificities. Written consent was obtained from all patients. This study was approved by the institutional review board of the University Claude Bernard Lyon 1 and Hospices Civils de Lyon.

**Table 1:** **Characteristics of patients included in the study**.

	CA	LE	SPS	Type 1 diabetes
**Other Autoimmune diseases**	Type 1 diabetes, Hashimoto	Hashimoto, Stiff Leg Syndrome	Type 1 diabetes	none
***N***	15	4	7	10
**Age**	60 (32–82)	44 (24–60)	45 (29–89)	12 (7–15)
**Gender**	11 F/4 M	4 F	7 F	4 F/6 M
**GAD65Ab titer (U/ml)**	6.0 × 10^5^ (3.9 × 10^4^–8.9 × 10^6^)	6.2 × 10^5^ (7.6 × 10^4^–1.5 × 10^6^)	1.1 × 10^6^ (2.5 × 10^5^–4.6 × 10^6^)	348 (341–1136)
**GAD65 binding in the presence of rFab b78 (%)**	81 (67–100	88 (79–94)	77 (66–81)	97 (87–99)
**GAD65 binding in the presence of rFab b96.11 (%)**	73 (44–94)	69 (58–99)	75 (55–78)	64 (42–85)

*Values are reported as median with range*.

### Monoclonal Antibodies Used in this Study

Human monoclonal antibodies b96.11 and b78 and mouse monoclonal antibody N-GAD65mAb specific to GAD65 were described before (Hampe et al., 2001; Manto et al., 2011). The antibodies were isolated by Protein G Sepharose from supernatants of the respective B cell lines or hybridoma and the protein concentration was adjusted to 1 mg/ml. Notably, only b78 inhibits the enzyme activity of GAD65 (Raju et al., 2005). Human monoclonal antibody HAA1 (ATCC Manassas VA, USA, ATCC number: HB-8534) is directed against Blood group A antigen and served as a control.

### GAD65Ab Radioligand Binding Assay and Epitope Mapping

GAD65Ab titers were determined by radioligand binding assay (RBA; Grubin et al., 1994; Bingley et al., 2003). The intra-assay coefficient of variation (CV) was 7.6%. In the International Combined Autoantibody Workshop, our assay showed 70% sensitivity and 98% specificity. The World Health Organization (WHO) standard (Mire-Sluis et al., 2000) was included as a standard to express immunoglobulin binding levels as a relative Unit (U/ml). The range of the standard curve was 30–1,000 U/ml. Samples that exceeded the upper end of the standard curve were titrated to half-maximal binding. Epitope mapping of GAD65Ab was performed on samples at half-maximal binding as previously described (Hampe et al., 2007). The cutoff for specific competition was determined as ≤15% as previously described (Hampe et al., 2007).

### Immunoisolation of GABAergic Synaptic Vesicles

Synaptic vesicles were prepared from whole rat brain as described by (Huttner et al. (1983). Briefly, synaptosomes were prepared by homogenizing fresh or frozen rat brain followed by a series of differential and sucrose-gradient centrifugation steps. Fractions containing the synaptic vesicle markers synaptophysin were pooled. Monoclonal antibody N-GAD65mAb crosslinked to Protein A Sepharose (PAS) was used to enrich for GABAergic vesicles. N-GAD65mAb-PAS was incubated with the pooled fractions for 2 h at 4°C while rotating. Bound immune complexes were washed extensively and analyzed for the presence of synaptophysin by Western blot utilizing a polyclonal anti-Synaptophysin rabbit antibody (Thermo Fisher Scientific, Rockford, IL, USA).

### Un-coupling of GAD65 from GABAergic Vesicles

To test whether b78 and/or b96.11 interfered with the association of GAD65 with GABAergic vesicles, we incubated the synaptic vesicle preparation with either monoclonal antibody (7 μg) for 1 h on ice. Human monoclonal antibody HAA1 was used as a negative control. After the initial incubation GABAergic vesicles were isolated as described above and the immunoprecipitated proteins were analyzed for the presence of synaptophysin.

### Patch-clamp Recordings of Cerebellar GABAergic Transmission

Experiments were performed on thin cerebellar slices from Sprague-Dawley rats (ages: 12–15 days, both sexes) as described previously (Saitow et al., 2005), in C57BL/6 WT mice and GAD65-KO mice back-crossed onto C57BL/6 (Yanagawa et al., 1999; Yamamoto et al., 2004) (ages: 14–18 days). Parasagittal slices of the brain (250 μm thickness) were superfused with artificial cerebrospinal fluid (ACSF) in 6-cyano-7-nitroquinoxaline-2, 3-dione (CNQX) (10 μM) (Tocris Cookson Bristol, UK) to eliminate glutamatergic excitatory synaptic responses.

Whole-cell voltage-clamp recordings held at −50 mV from Purkinje cells (PCs) were performed as previously described (Saitow et al., 2005). Individual slices were transferred to a recording chamber attached on the stage of a microscope (BX61WI, Olympus, Japan) and continuously perfused with oxygenated ACSF at a flow rate of 1.3–1.5 ml/min at 25–27°C. Monoclonal antibodies (diluted to 10 μg/ml in ACSF) were applied by perfusion for 10 min. For recording GABAergic miniature inhibitory postsynaptic currents, the cells were voltage clamped at −20 mV in order to increase electromotive force for GABAergic currents. Responder cells were defined as showing Ab-induced inhibition 3 min after the bath application, which lasted for more than 20 min and an inhibition rate >20% (percent changes in the IPSC before and after was below 80%). All animal experiments were approved by the Ethics Review Board of Nippon Medical School.

### Eyeblink Conditioning

Experiments were carried out on 3 months old, male C57BL/6 mice (University of Seville Animal House, Seville, Spain). All of the experiments were carried out in accordance to European Union (2003/65/CE) and Spanish (BOE 252/34367-91,2005) guidelines for the use of laboratoryanimals in chronic experiments and were approved by the Ethical Committee of the Pablo de Olavide University.

#### Surgical Preparation for Classical Eyeblink Conditioning

Anesthetized mice were implanted with two EMG recording electrodes in the left orbicularis oculi muscle and a pair of recording electrodes aimed to the ipsilateral supraorbital nerve. Electrodes were made of Teflon-insulated, annealed stainless steel wire (50 μm in diameter, A–M Systems, Carlsborg, WA, USA). An electrode was fixed to the cranial bone as ground. Electrodes were connected to a 6-pin socket (RS-Amidata, Madrid, Spain), which was fixed with dental cement to the cranial bone. Animals were also implanted with a C315GS-4 stainless steel cannula (Plastic One, Reanoke, VA) on the ipsilateral interpositus nucleus (1.2 mm lateral and 6.36 mm posterior to bregma and 3 mm from brain surface, i.e., 0.25 mm above the infusion target (Paxinos and Franklin, 2001) for b78 administration. After surgery, animals were kept in independent cages, with free access to food and water, and allowed proper recovery (1 week) before the beginning of the experiment.

#### Classical Conditioning Procedures of Eyelid Responses

Classical conditioning of eyelid responses were performed as previously described (Guerra-Narbona et al., 2013), using 10 animals per group and trace and delay conditioning paradigms. Briefly, a short (for trace) or long (for delay) tone was used as conditioning stimulus (CS), whilst an electrical shock applied to the supraorbital nerve (presented 250 ms from CS start) was used as unconditioned stimulus (US). Conditioned responses were determined from the EMG activity evoked in the orbicularis oculi muscle during the CS-US interval. The rectified area of EMG activity during the same interval was used to determine eyelid performance during conditioned eyeblinks (Domínguez-del-Toro et al., 2004; López-Ramos et al., 2010). A total of one habituation, five conditioning, and one extinction sessions (120 trials each) were presented to each animal in seven successive days. Only the first fifty trials of each session were analyzed (Gruart et al., 2006; López-Ramos et al., 2010). Prior (30 min) to each conditioning and extinction session, b78 (1.5 μg) or saline (1.5 μl) was administered through an injection cannula. The injection cannula was 250 μm longer than the implanted cannula, which was used as a guide. Location of the implanted cannula was verified as described previously (Gruart et al., 2012). Distribution of infused GAD65Ab in the deep cerebellar nuclei was confirmed by immunohistochemistry on cerebellar slices.

#### Data Collection and Analysis of Eyelid Responses

Data were recorded and analyzed as described previously (Guerra-Narbona et al., 2013). Criteria for conditioned responses were as before (Domínguez-del-Toro et al., 2004; López-Ramos et al., 2010; Guerra-Narbona et al., 2013). EMG and 1-V rectangular pulses corresponding to CS and US presentations were stored digitally on a computer through an analog/digital converter (CED 1401 Plus, Cambridge, England). Collected EMG data were quantified through a purpose-designed Excel worksheet, as the percentage of conditioned responses per session—i.e., the proportion of paired CS-US stimulations within a session of 50 presentations that generated an EMG activity satisfying the above-mentioned criteria (Domínguez-del-Toro et al., 2004; López-Ramos et al., 2010). In addition data were analyzed off-line for quantification of rectified EMG area (μV × s) with the Spike 2 (CED) program.

### Modulation of Corticomotor Excitability

Experiments were approved by the Animal Care Committee of ULB. Rats were bred in the local animal care facility and received water and food *ad libitum*.

#### Infusion of Ab

Adult anesthetized Wistar rats (weight: 206–420 gr, 25 males, 22 females) were implanted with guides (CMA12, CMA, Sweden) in the left cerebellar interpositus nucleus (coordinates related to bregma: −11.6, L: +2.3, V: −4.6 (Paxinos and Watson, 2007). A needle (Hamilton point style 4, Hamilton) was inserted in the guide. The extremity of the needle was located 0.2 mm below the extremity of the guide. Monoclonal antibodies or controls (total volume: 5 μl of 1 mg/ml) were infused using a micropump (CMA100, CMA, Sweden) at a flow rate of 1 μL/min. Animals were infused with Ringer solution (*n* = 9) (Group 1), b96.11 (*n* = 10) (Group 2), or b78 (*n* = 7) (Group 3). A fourth group of four rats was infused with b78 in the fastigial nucleus (coordinates related to bregma: −11.8, L: +0.5, V: −5.4), to assess the effect of the zone injected in the cerebellum.

To exclude local bleeding following the experimental procedures, we monitored the local blood flow at the beginning and end of the experiments using laser flowmetry (Oxylab, Oxford Optronix). A laser flow probe was inserted near the tip of the guide to monitor the blood flow locally in the brain (microvascular perfusion). This technique allows the early detection of bleeding (immediate drop in blood flow) and was validated in 12 rats. Blood flow rates (expressed in arbitrary units BPU (blood per units) allowing evaluation of relative changes in perfusion; see Tonnesen et al., 2005) at the beginning and end of each experiment were determined. Rats with impaired blood flow were excluded from the analysis.

#### Analysis of Corticomotor Excitability

Muscle recordings were performed as previously described with minor modifications (Hosoido et al., 2009). For forelimbs and hindlimbs, we analyzed the corticomotor responses evoked in interosseus muscles on the left side following stimulation of the right motor cortex (Oulad Ben Taib et al., 2005). We used subcutaneous electrodes (Technomed 017K025) implanted in interosseus muscles. The impedance was kept below 5 KΩ.

In previous studies, we determined the “hot spot” of the gastrocnemius muscle (Oulad Ben Taib et al., 2005). This allowed us to identify the precise location corresponding to the largest motor evoked potential (MEP; confirmed by epidural stimulation with tungsten microelectrodes TM33A05, World Precision Instruments, UK), which was found to be located between 2–4 mm laterally, and between 1 mm anterior and 2 mm posterior (coordinates related to bregma). A similar methodology for mapping of MEPs (identification of the hot spot for each muscle) was applied here. The duration of stimuli was 1 ms (square waves; NeuroMax 4, Xltek, Canada). Recruitment curves (detection of motor threshold MT defined as the lowest intensity eliciting at least five out of 10 evoked responses with an amplitude >20 μV, followed by increases of the intensity of stimulation with steps of 0.1 mA until a plateau) of corticomotor responses were analyzed to confirm the classical sigmoid course using a sigmoid fitting with three parameters: *y* = *a*/(1 + *exp*(−(*x*−*x*0)/*b*)) (Oulad Ben Taib et al., 2005). Subsequently, motor cortex was stimulated at an intensity of 130% of MT to assess corticomotor potentials. Peak-to-peak amplitudes in motor responses of the left interosseus muscles were studied for groups of 10 successive corticomotor responses (Filter settings were 30 Hz–1.5 kHz; NeuroMax 4, Xltek, Canada).

#### Paired-Pulse Corticomotor Responses

At a short latency between a peripheral nerve stimulus and a second stimulus (called test stimulus) applied contralaterally on motor cortex, the corticomotor response is increased due to facilitation (pairing effect) (Oulad Ben Taib et al., 2005; Oulad Ben Taib and Manto, 2008). To evaluate these paired-pulse corticomotor responses, test stimuli on right motor cortex (NeuroMax 4, Xltek, Canada) were preceded by a first inter-stimulus interval (ISIs) of 4 ms and in contralateral sciatic nerve (for hindlimbs) at an ISI of 6 ms (Oulad Ben Taib and Manto, 2008). For forelimbs, we used ISIs of 10 and 14 ms in three rats to confirm our observations and to exclude a contribution of a pure spinal facilitation.

#### Trains of High-Frequency Stimulation (HFS) Over rFr2

Trains of stimulation were applied in right prefrontal region rFr2 as described previously (Oulad Ben Taib and Manto, 2008) with minor modifications. Trains of HFS consisted in 25 successive rectangular pulses, with a pulse duration of 500 μs and an intra-train interval between two successive pulses of 1 ms (1000 Hz). Trains were repeated every 50 ms for a period of 14 min. We studied here the effects of premotor HFS on the paired-pulse corticomotor responses.

Corticomotor responses were analyzed at baseline (a) without (unpaired motor responses UMR); and (b) with a preceding stimulus (paired-pulse motor responses CMR). Following infusion in cerebellar nuclei, corticomotor responses were re-assessed between 30 and 45 min after infusion without and with preceding stimulus (to evaluate the infusion effect). Trains of HFS were applied subsequently and the corticomotor responses were re-assessed immediately (at a timing of ~65–80 min after infusion) after, both without and with a pairing stimulus immediately after (HFS effect) (Manto et al., 2011).

### Behavioral Data

We assessed the effects of intra-nuclear administration of GAD65Ab bilaterally upon the exploration behavior (open field) and upon locomotor activity (beam test). Rats were assessed 6 h after the administration of GAD65Ab, using two digital cameras (Canon ZR40). Displacement times and number of squares crossed were obtained by extracting manually the position of rats from digitally reconstructed images using the APAS system (Ariel Dynamics, USA). The images were filtered using a Sobel filter providing contrasted pictures by emphasizing edges and transitions. The spatial accuracy was 1.5 mm. The sampling rate was 60 Hz. The reproducibility of the method has been assessed with 2 independent observers (beam test: Pearson product moment correlation = 0.958; open field: Pearson product moment correlation = 0.974). For the open field test (square open field of 68 × 68 × 29 cm; divided in 25 identical squares), rats were placed at the center of the open field at the beginning of the procedure. They were first left undisturbed for 40 min before testing. The number of squares crossed over a period of 3 min was counted. Animals in groups of five rats were infused with b78 (Group 1), b96.11 (Group 2), Ringer solution (Group 3), or no injection (sham procedure: anesthesia with chloral hydrate, surgical opening of the skin immediately followed by suture) (Group 4). Five trials were performed for each rat. The arena was cleaned carefully between each measurement. For the beam test, rats had to traverse an elevated horizontal beam of 80 cm. We counted the displacement time from a starting box to a goal box. During a short training period (10 trials), the animal was repeatedly placed on the beam in the starting box to move to the goal box without interruption.

#### Histological Verification

At the end of the experiments, brains were extracted from the skull to exclude a traumatic lesion (Manto et al., 2011). We assessed brain sections similar to a method previously published (Bert et al., 2004). Only data with correct location of the guide (the extremity of the guide located in the target region) were analyzed.

#### Statistical Analysis

Differences in GAD65Ab titer and epitope specificities were determined using Kruskal-Wallis test. The extent of the inhibitory effect of each antibody in the cerebellar slide studies were compared using the Tukey-Kramer multiple comparison test. Cumulative curves were compared using the Kolmogorov-Smirnov (K-S) test. Regarding the modulation of the excitability of corticomotor responses in rats, for both forelimbs and hindlimbs, we applied an analysis of variance to compare the amplitudes of MEPs in the groups 1–3 at baseline before paired stimulation (in order to exclude differences between the three groups at the beginning of the experiments). For MEPs in both forelimbs and hindlimbs, we assessed the effects of the paired-pulse procedure (ratios CMR/UMR = ratios paired corticomotor responses/unpaired corticomotor responses) in the three groups and in the three states (basal state, post-injection and post-HFS) using the analysis of variance with post-hoc multiple comparison procedures (Bonferroni test). For behavioral tests (open field, beam test), we applied the Kruskal-Wallis one way analysis of variance on ranks followed by a pairwise multiple comparison procedures (Tukey-Kramer test).

## Results

### GAD65Ab Epitope Specificities in Autoimmune Movement Disorders

**Figure 1:**
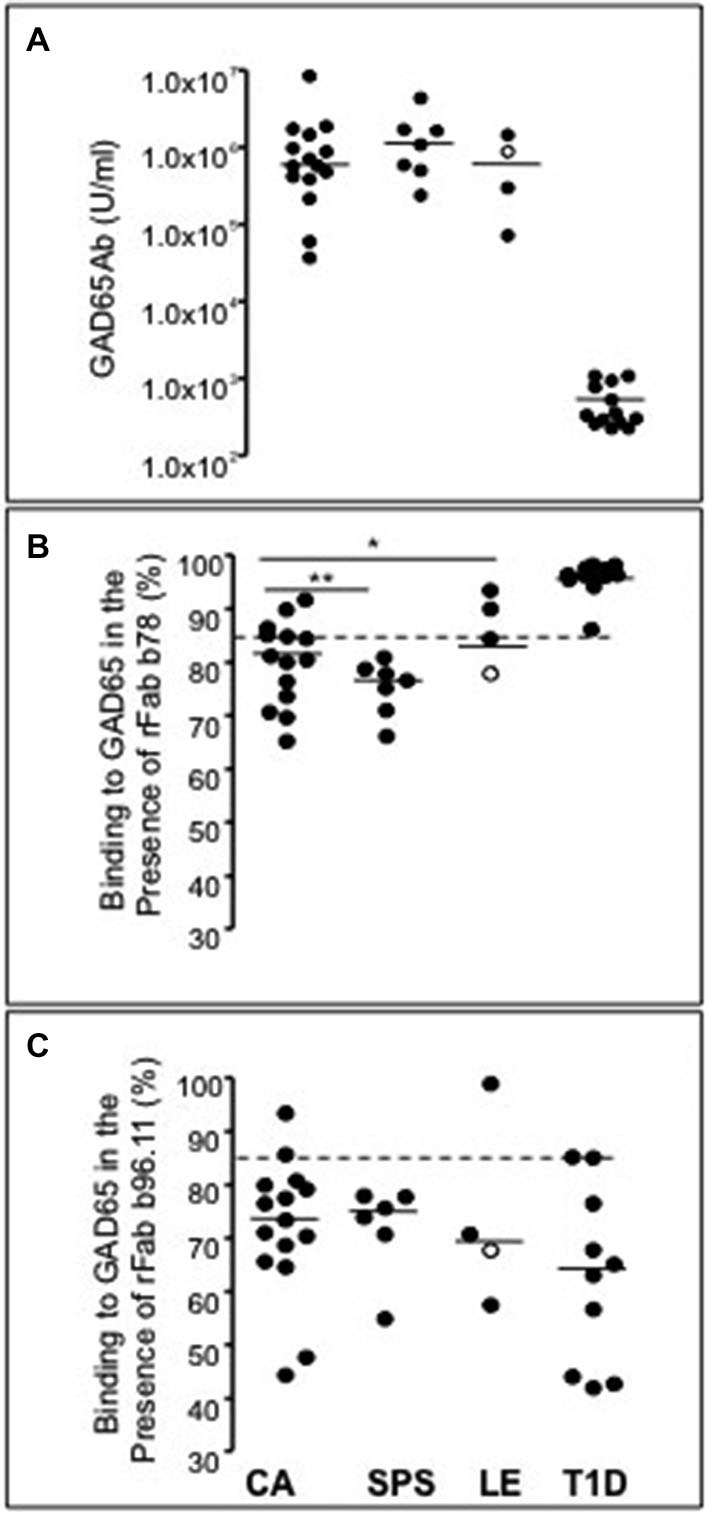
**GAD65Ab titers and epitope specificities in patients with neurological disorders**. GAD65Ab titers **(A)** and epitope specificities **(B,C)** in patients with SPS, cerebellar ataxia, SPS, LE, and type 1 diabetes patients without neurological symptoms were evaluated. Results for the LE patient that presents also stiff-leg syndrome are indicated as an open circle. GAD65Ab titers are presented as U/ml. Binding to GAD65 in the presence of rFab is presented as percent related to non-competed binding (set as 100%). Median binding is indicated. Significant differences are shown. Cut-off value for significant competition of GAD65Ab binding by recombinant Fab is indicated as the dotted line in panels **(B,C)**. * = *P* < 0.05, ** = *P* < 0.01.

Our analysis of GAD65Ab epitope specificities and titers in patients with different neurological syndromes, revealed no significant differences in GAD65Ab titers (Figure 1A) or in recognition of the b96.11-defined epitope (Figure 1C). However patients with SPS recognized the b78-defined epitope significantly better than patients with ataxia (*P* = 0.03) or LE (*P* = 0.01) (Figure 1B). Patients with ataxia showed higher recognition of the b78-defined epitope than patients with LE, however this difference was not significant (*P* = 0.07). Notably, one LE patient was diagnosed with stiff-leg syndrome. This patient showed the strongest recognition of the b78-defined epitope in the LE group. Ten patients with T1D were included in our analysis to confirm our previous finding that GAD65Ab in T1D patients show preferred binding to the b96.11-defined epitope, while lacking binding to the b78-defined epitope (Padoa et al., 2003).

### Co-localization of GAD65 with GABAergic Vesicles is Interrupted by b78

**Figure 2:**
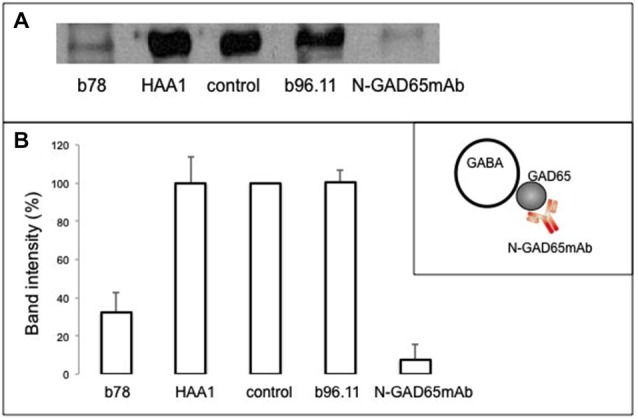
**Effect of GAD65Ab on association of GAD65 with GABAergic vesicles**. Synaptic vesicles were incubated with monoclonal antibodies b78, HAA1, b96.11, or N-GAD65mAb for 1 h on ice. No antibody was added for the control sample. GABAergic vesicles were isolated as outlined in the inset sketch and the immunoprecipitated proteins were analyzed for the presence of synaptophysin by Western blot. **(A)** Representative Western blot analysis. **(B)** Densiometric analysis from three independent experiments. Results are presented as percentage of the corresponding control sample. Error bars represent the standard deviation of the mean. Inset: schematic representation of the experimental set-up.

To establish whether GAD65Ab interfere with the association of GAD65 and GABAergic vesicles, we immunoprecipitated GABAergic vesicles in the presence of GAD65Ab. Synaptic vesicles were isolated from rat brain and incubated with or without b78, b96.11, or HAA1. GABAergic vesicles were precipitated with monoclonal antibody N-GAD65mAb. Immune complexes were analyzed by Western blot for the presence of synaptophysin (Figure 2). Precipitation of GABAergic vesicles by N-GAD65mAb-PAS was abolished through competition with uncoupled N-GAD65mAb, showing specificity of the immune precipitation. We found a significant reduction of the synaptophysin signal in the presence of b78, while b96.11 and HAA1 showed no effect. These results suggest that b78 interferes with the association of GAD65 to GABAergic vesicles. Previously we established that binding of monoclonal antibody N-GAD65mAb did not interfere with binding of b96.11 or b78 to GAD65 (Fenalti et al., 2008), ensuring that the observed results were not caused by displacement of N-GAD65mAb from GAD65.

### *In Vitro* Analysis of the Effects of b78 and b96.11 on Cerebellar GABAergic Inhibition

**Figure 3:**
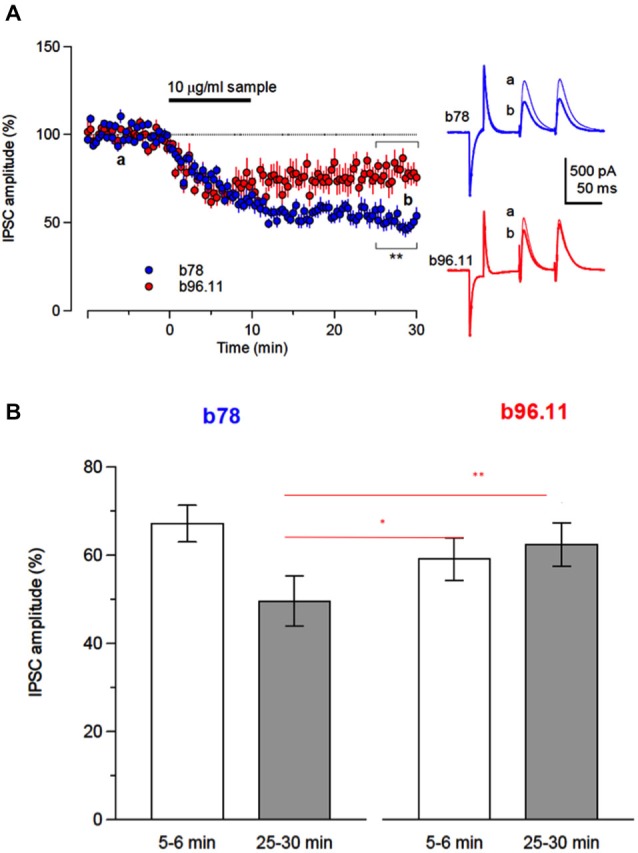
**Inhibition of GABAergic synaptic transmission by monoclonal GAD65Ab. (A)** Time-course of changes in evoked IPSC amplitude by b78 (blue circles, *n* = 12) and b96.11 (red circles, *n* = 9) in cerebellar Purkinje cells. Each point represents the mean (±SEM). Inset: Responses are shown as averages of ten consecutive sweeps recorded before (a, black lines) and after (b, blue line for b78 and red line for b96.11) application of diluted-antibodies, respectively. Traces in a and b were obtained at the time points indicated in the left panel. **(B)** Summary of the antibody-induced suppressive effects on the inhibitory synaptic transmissions. To compare the effects of monoclonal antibodies on the inhibitory transmission, we set two time windows that covered early onset of inhibition (5–6 min, open bars) and sustained-inhibition after wash-out of antibodies (25–30 min, closed bars). Asterisks indicate the significance level compared with each groups by Tukey-Kramer multiple comparison test. Each point represents mean ± SEM.

Focal stimulation within the molecular layer in the cerebellar cortex produces inhibitory postsynaptic currents (IPSCs) in Purkinje cells, which are mediated by GABA released from basket cells. Responder cells were defined as showing Ab-induced inhibition 3 min after the bath application, which lasted for more than 20 min and an inhibition rate >20% (percent changes in the IPSC before and after was below 80%). While application of either b78 or b96.11 suppressed the amplitude of IPSCs in 12/24, and 9/26 tested cells, respectively (Figure 3), the time-course of the synaptic depression in responder cells differed considerably between the antibodies (Figure 3A). Administration of b78 resulted in a gradual decrease of the IPSC amplitude, beginning 5–6 min after the application (mean: 67.2 ± 4.2% of the control), and reaching a final decrease of 49.6 ± 5.7% compared to the control, which lasted for more than 30 min. Reduction of the IPSC amplitude following administration of b96.11 occurred relatively faster, reaching 65.7 ± 5.8% of the control already 5–6 min after the application, however, the inhibitory effects tended to attenuate and the amplitude recovered to 78.0 ± 6.1% of the control after 25–30 min. Therefore no significant differences were observed in the short-term reduction of IPSC amplitude (5–6 min after antibody administration) between in slices perfused with b78 and in slices perfused with b96.11 (67.2% and 65.7% and 67.2%, respectively, *P* = 0.95), whereas the long-term effect (25–30 min after antibody administration) of b78 resulted in a significantly stronger reduction in IPSC amplitude, as compared to b96.11 (49.6% and 78.0%, respectively, *P* = 0.003; Figure 3B).

**Figure 4:**
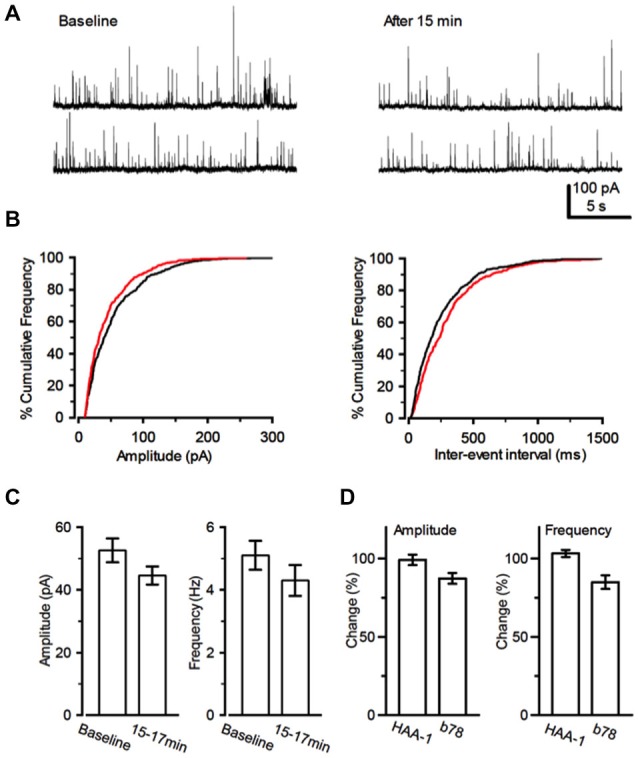
**Effect of b78 on miniature IPSCs. (A)** An example of changes in miniature IPSCs (mIPSCs). mIPSCs were recorded from a PC before (left) and 15 min after the application of b78. **(B)** Cumulative curves for the amplitude (left) and the inter-event intervals (right) distributions of mIPSCs were compiled from 2 min records before (black lines) and after (red lines) the b78 application, respectively. **(C)** Summary data for the effects of b78 on the mIPSC amplitude (left; Baseline, 52.6 ± 3.8 pA; 15–17 min; 44.6 ± 2.9 pA; *t*_11_ = 2.67, *P* = 0.02, *n* = 12) and frequency (right; Baseline, 5.1 ± 0.5 Hz; 15–17 min; 4.3 ± 0.5 Hz; *t*_11_ = 3.65, *P* = 0.004, *n* = 12) in PCs. Each column represents mean ± SEM that is calculated from individual mIPSC events for 2 min before (baseline) and 15 min after the b78 application in 12 tested cells. **(D)** The extent of change of the amplitude (left) and the frequency (right) of mIPSCs by b78 (*n* = 12) was compared with that observed after administration of control HAA1 (*n* = 12). The percent of change was calculated by the proportion of the value after 15–17 min to that before the application of each monoclonal Ab.

The ratio of the magnitude of the second IPSC to that of the first IPSC to paired-pulse stimuli was defined as the paired-pulse ratio (PPR). During the inhibitory period caused by both antibodies, PPR was increased, suggesting a presynaptic mechanism. Miniature IPSCs (mIPSCs) were analyzed to elucidate changes underlying presynaptic depression. Application of b78 decreased the amplitude of mIPSCs and reduced frequency in most of the tested cells (*n* = 12) (Figures 4A,B). Thus, the statistical analysis was calculated from all tested cells. The cumulative curve of the amplitudes was significantly higher after b78 application (Kolmogorov-Smirnov test; *P* = 0.01). Correspondingly, the cumulative curve of the inter-event intervals was significantly lower after b78 application (Kolmogorov-Smirnov test; *P* = 0.007). After the perfusion of b78, the amplitude decreased to 87.0 ± 3.5% of the control (*n* = 12) and the frequency was reduced to 84.7 ± 4.4% of the control (*n* = 12) (Figure 4C). Control monoclonal Ab HAA1 produced no effect on miniature IPSCs (*n* = 12) in amplitude or frequency (99.1 ± 3.4% and 103.2 ± 2.3% of the controls, the mean and SEM 15–17 min after the application, respectively). Amplitude and frequencies observed after application of b78 were significantly lower when compared to those recorded after application of HAA1 (Amplitude: *t*_22_ = 2.39, *P* = 0.03; Frequency: *t*_22_ = 3.68, *P* = 0.001, Figure 4D). These results supported our hypothesis that monoclonal GAD65Ab reduce GABAergic inhibition via reduced concentrations of presynaptic released GABA.

### GAD65 Knockout Mice

The above evaluations were also performed in wild-type C57BL/6 mice (WT) and GAD65-knockout mice (GAD65-KO). Using the same criteria as in the rat experiments we defined responder and non-responder cells in the WT mice. Numbers of responder and non-responder cells in WT and GAD65-KO are shown in Table 2. Six out of 14 tested cells met the criteria of responder cells. Importantly, none of the nine cells studied from GAD65-KO met the criteria of responder cell (Table 2).

**Table 2:** **Actions of monoclonal Ab b78 on stimuli-evoked IPSCs**.

	Responder cells	Non-responder cells	Total cells analyzed
Wild-type	6	8	14
GAD65 knockout	0	9	9
Sprague-Dawley rats	12	12	24

*Numbers of responder and non-responder cells are reported in studies of cerebellar slices obtained from WT and GAD65-KO mice, and rats, respectively*.

**Figure 5:**
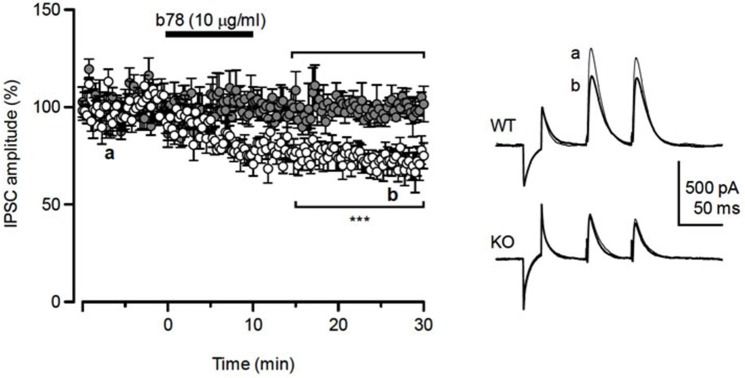
**Response of GAD65-KO mice to b78 injection**. Left panel: Mean IPSC amplitude changes of the six responder cells in WT (open circles) and all tested cells in GAD65-KO (gray circles). Significant differences were observed between the two groups during the 15–30 min after b78 applications, (*p* = 1 × 10^−4^). Right panel: Responses are shown as averages of eight consecutive sweeps recorded before (a, thin lines) and after (b, thick lines) application of diluted-antibody, respectively. Traces in a and b were obtained at the time points indicated in the left panel.

Compared to cells from GAD65-KO, the six WT responder cells showed significantly lower IPSC amplitude changes during the 15–30 min period following b78 application (*t*_13_ = −5.68, *p* = 1 × 10^−4^) (Figure 5). These results indicate that while b78 suppressed IPSCs in WT, similar to our results in rats, GAD65-KO cells show no response to b78 injection.

### Classical Conditioning in Mice

Classical eyeblink conditioning using both trace and delay paradigms was performed in non-injected mice and in animals injected with b78. During the trace paradigm, all animals showed evidence of learning, as CR percentages increased progressively over time. Control animals showed 33.6 ± 6.6% (mean ± SEM, *n* = 10) CRs on the 1st conditioning day, and reached peak values by the 4th conditioning session (43.7 ± 5.1%). Mice injected with b78 showed 29.2 ± 5.9% during the 1st conditioning session and 44.5 ± 4.8% during the 3rd. No significant differences in CR percentages or relative amplitude of the evoked CR for the two groups were detected (data not shown). Moreover, no significant differences were detected when comparing the conditioned responses in animals receiving b78 and control animals (data not shown).

**Figure 6:**
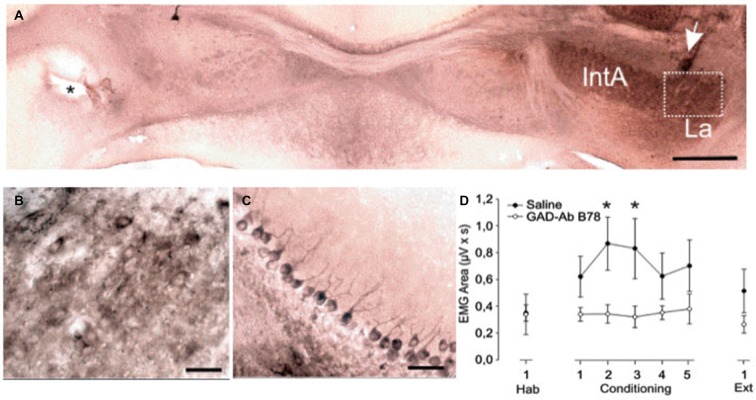
**Learning curves and eyelid performance collected from controls (saline) and experimental (b78) mice during a delay conditioning paradigms**. Spreading of b78 in the deep cerebellar nuclei was confirmed by immunohistochemistry. **(A)** Presence of b78 in deep nuclei areas of the right cerebellar hemisphere. The injection site in the contralateral area is indicated by the asterisk. **(B)** Enlargement of the marked area in panel **(A). (C)** Purkinje cells of a lobule area proximal to the injection site. Abbreviations: IntA, anterior part, La, lateral part. Calibration bars: **(A)**: 200 μm, **(B,C)** 50 μm. **(D)** Conditioned eyelid responses evoked in controls were significantly larger (*F*_(1,14)_ = 4.6; *P* = 0.05; see asterisks) than those evoked in b78 animals.

However, significant differences were observed for the area of rectified EMGs collected at the CS-US interval during delay conditioning (Figure 6). Here conditioned eyelid responses evoked in controls were significantly larger as compared to those in mice administered with b78 (*F*_(1,14)_ = 4.6; *P* < 0.05).

### Modulation of Corticomotor Responses in Rats

**Figure 7:**
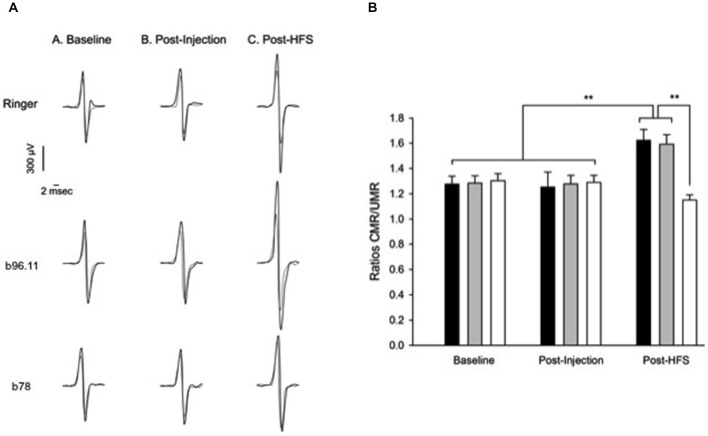
**Effect of GAD65Ab on conditioned corticomotor responses and on motor evoked potentials illustrated by ratio of paired/unpaired corticomotor responses. (A)** Superimposition of averaged motor evoked potentials (MEPs) obtained without preceding peripheral stimulation (unpaired responses, thin traces) and with a pairing stimulus (thick trace). Pairs of corticomotor responses are shown at baseline (before injection; traces on the left), after injection (middle traces), and after high-frequency stimulation (post-HFS; traces on the right). From top to bottom: infusion of Ringer solution, infusion of b96.11, and infusion of b78. Averaged MEPs in left interosseus muscles (forelimbs) are illustrated (stimulation of right motor cortex). Note the marked increase in the intensity of paired responses in forelimb after HFS for the rat injected with Ringer solution and the rat injected with b96.11. By contrast, b78 induces a striking block of the facilitatory effect normally exerted by HFS of the premotor cortex upon conditioned corticomotor responses. **(B)** Effects of GAD65Ab injection in left interpositus nucleus on the ratios of paired corticomotor responses (CMR) divided by unpaired responses (UMR) in Groups 1–3. Data related to MEPs in left interosseus muscles (forelimbs) are illustrated. Black columns: infusion of Ringer solution (*n* = 9); gray columns: infusion of b96.11 (*n* = 10); white columns: infusion of b78 (*n* = 7). Paired responses are investigated at baseline, post-injection and after high-frequency stimulation (post-HFS). Values are mean ± SD. **P < 0.01.

Next we determined whether the interference of monoclonal GAD65Ab on GABA neurotransmission had an effect on the electrophysiology. We infused rats in their cerebellum with Ringer (Group 1), b96.11 (Group 2) or b78 (Group 3). Corticomotor responses evoked in interosseus muscles following stimulation of the contralateral motor cortex were recorded. In Groups 1–3, latencies of corticomotor responses in interosseus muscles were between 5.1 ms and 6.7 ms in forelimbs and between 8.3 and 9.4 ms in hindlimbs. The range of peak-to-peak intensities of M responses was between 3.6 and 6.5 mV (Figure 7A). At baseline, amplitudes of MEPs were similar in all groups for forelimbs (range of intensities: 659–884 μV) and hindlimbs (range of intensities: 673–872 μV) (Figure 7A, Baseline). Similarly, no differences were observed after injection (Figure 7A, Post-injection). However, after application of trains of stimuli (HFS) over the premotor cortex, rats injected with Ringer or b96.11 showed marked facilitation of the pairing effect following HFS (Figure 7A, Post-HFS). By contrast, injection of b78 induced a striking block of this facilitation. A similar observation was made for hindlimbs (data not shown). In addition, using ISIs of 10 and 14 ms between the medialis nerve and the contralateral motor cortex led to similar findings (data not shown).

For MEPs in forelimbs, the effect of the paired-pulse procedure (ratios CMR/UMR = ratios paired corticomotor responses/unpaired corticomotor responses) was assessed in the three treatment groups and in the three states (basal state, post-injection and post-HFS) (Figure 7B). A conditioning effect was found for all three groups of rats in all states (*P* < 0.001; Bonferroni test: *P* < 0.05). However, the enhancement of the pairing effect associated with HFS of the premotor cortex was significantly higher in animals injected with Ringer solution or b96.11 as compared to animals injected with b78 (*P* < 0.001).

In the basal condition, the mean ratios CMR/UMR were similar for animals injected with Ringer solution, b96.11, or b78 (1.28 ± 0.06, 1.24 ± 0.06 and 1.26 ± 0.05, respectively). After injection, no significant differences in the mean ratios CMR/UMR were observed between the groups (1.27 ± 0.05, 1.30 ± 0.04 and 1.25 ± 0.04, respectively). However, after HFS, the mean ratios CMR/UMR for animals injected with Ringer solution or b96.11 were significantly higher (1.50 ± 0.07, 1.52 ± 0.05, respectively), as compared to animals administered with b78 (1.15 ± 0.04) (inter-group difference: *P* < 0.001). Similar observations were made for the hindlimbs (data not shown).

In rats administered with b78 in the fastigial nucleus, the facilitation of the pairing effect following HFS was very similar to the values observed in animals injected with Ringer solution or b96.11. For forelimbs, the mean ratios CMR/UMR (± SD) were 1.30 ± 0.06 in the basal condition, 1.33 ± 0.07 post-injection and 1.59 ± 0.08 post-HFS. For hindlimbs, the mean ratios CMR/UMR (± SD) were 1.28 ± 0.07 in the basal condition, 1.29 ± 0.05 post-injection and 1.55 ± 0.07 post-HFS.

### Behavioral Data

**Figure 8:**
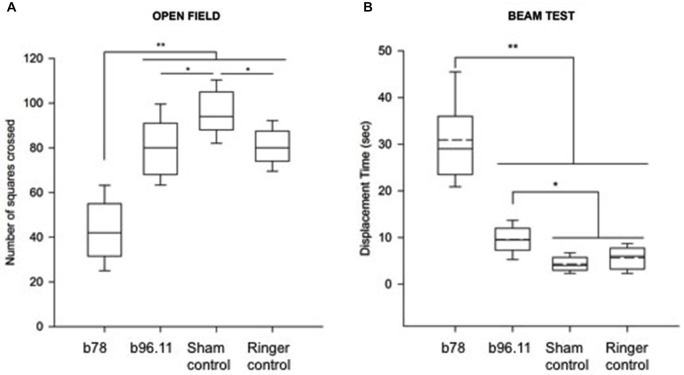
**Effect of GAD65Ab on behavior of rats as assessed in open field test and elevated beam test. (A)** Effects of bilateral administration of GAD65Ab in the interpositus nucleus on exploration behavior in rats (open field test). Four groups of five rats were assessed. The number of squares crossed was measured over a period of 3 min (five trials per rat). Box plots showing median values, p25 and p75 with outliers. **P < 0.001, *P < 0.05. **(B)** Displacement times (expressed in seconds) during the horizontal beam test. Four groups of three rats were assessed. Median with mean values (dotted lines) are shown, as well as p25/p75 and outliers. **P < 0.001, *P < 0.05.

Effects of GAD65Ab on exploration behavior (open field) and locomotor activity (beam test) were assessed. For exploration behavior the number of squares crossed in an open field by rats infused with b78, b96.11, Ringer solution, or non-injected animals was analyzed (Figure 8). The administration of b78 decreased the exploration behavior of rats in the open field significantly as seen in the number of squares crossed compared to the other groups (*P* < 0.001). Gait was moderately irregular. Both groups injected with b96.11 and Ringer solution showed a slight but significant decrease in exploration behavior as compared to the group without injection (*P* < 0.05), however the animals’ gait was regular.

For assessment of locomotor activity the animals had to traverse an elevated horizontal beam (beam test). Displacement times were counted. Rats administrated with b78 showed significantly greater displacement times compared to the other groups (*P* < 0.001). Moreover, their locomotion was slower, with foot slips but without falling. In addition, we also found that b96.11 slightly increased the displacement times as compared to the sham group (no infusion) and the group infused with Ringer solution (*P* < 0.05).

## Discussion

We now provide critical findings for the understanding of the pathogenesis of GAD65Ab. The observation of disease-specific GAD65Ab in neurological disorders led to the hypothesis that GAD65Ab target and inhibit GAD65 in these diseases. Passive transfer experiments supported this hypothesis (Geis et al., 2011; Manto et al., 2011; Hampe et al., 2013; Hansen et al., 2013; Vega-Flores et al., 2014). Importantly, IgG from patients’ CSF suggested that GAD65Ab acted on synaptic transmissions in an epitope-dependent manner, which may result in the development of neurological symptoms. IgG from ataxia patients depressed the cerebellar inhibitory synaptic transmissions in slices (Ishida et al., 1999; Mitoma et al., 2003) and impaired the cerebellar modulation on the motor cortex (Manto et al., 2011). In contrast, IgG from patients with T1D had no effect on synaptic transmissions or modulation on the motor cortex (Ishida et al., 1999; Mitoma et al., 2003; Manto et al., 2011). These effects were confirmed *in vivo* (Geis et al., 2011; Manto et al., 2011; Hampe et al., 2013; Hansen et al., 2013; Vega-Flores et al., 2014). However, to establish a pathogenic role for GAD65Ab, we need to address (a) The mechanism by which GAD65Ab inhibit GABAergic neurotransmission, e.g whether the depression occurs presynaptic or postsynaptic; and (b) The ability of GAD65Ab to elicit deficits in cerebellar coodination on reflexes, gait, and limb movements *in vivo*. To address these critical problems, we conducted the further advanced analysis using monoclonal GAD65Abs in the present study. Moreover, we addressed the possibility that the observed effects are caused by the binding of GAD65Ab to proteins other than GAD65.

Our study utilized two well-defined monoclonal GAD65Ab to focus on epitope-specific effects of GAD65Ab and to eliminate the impact of other factors present in patients’ sera. The specificity of GAD65Ab b78 is associated with SPS, while that of GAD65Ab b96.11 is associated with T1D and only rarely found in patients with SPS (Raju et al., 2005; Manto et al., 2007). These previous findings were now confirmed and extended in our analysis of GAD65Ab epitope patterns in GAD65Ab-positive neurological disorders. SPS and ataxia patients showed strong recognition of the b78-defined epitope, while LE patients showed no significant recognition of this epitope. The presence of b96.11-like GAD65Ab in sera of SPS and CA patients is likely due to the co-diagnosis of T1D in many of these patients. Overall, the GAD65Ab response in SPS and CA patients is skewed towards a b78-like epitope specificity, while that in T1D patients is skewed towards a b96.11-like epitope specificity. A more specific identification of the GAD65Ab epitope is hindered by the conformational characteristic for autoepitopes (Mackay and Rowley, 2004).

Our investigation of the mechanisms involved in the GAD65Ab-mediated pathogenesis suggests a b78-specific interference with GABAergic synaptic transmission. Cerebellar slice experiments suggested an inhibitory effect of GAD65Ab on the GABAergic synaptic transmission between basket cells and Purkinje cells, with distinct effects of b78 and b96.11. GAD65Ab b78 depressed the inhibitory synaptic transmission with a gradual time course and lasting suppressive effect, while b96.11 caused only a transient depression of transmission. Previous studies using slice-patch recordings have shown that GAD65Ab in ataxia patients act on the terminals of GABAergic neurons to suppress the release of GABA, thereby depressing the inhibitory transmission with a gradual and long-lasting time course (Ishida et al., 1999; Mitoma et al., 2003). The synaptic impairment by GAD65Ab in patients’ CSF was mimicked by b78 and not b96.11. Our observation of the decrease in GABA release by the PPR analysis indicates that b78 may affect a presynaptic mechanism that induces this synaptic impairment.

In contrast to b78, b96.11 affected neither GAD65 enzyme activity nor the exocytosis of vesicle (Padoa et al., 2003; Manto et al., 2007). Although b96.11 elicited a temporal inhibition in our transmission experiment, the intra-cerebellar administration of b96.11 had no effect on the activity in the cerebello-cerebral circuits (Manto et al., 2011). Thus, the inhibition by b96.11 in slices appears to have no significant physiological effects due to its temporal nature. On the other hand, both b78 and b96.11 produced the same early inhibition in the time-course of percentage changes in IPSCs (see Figure 3). Thus, the early component induced by both Abs in slices might have no physiological significance *in vivo*, whereas the late component specific to b78 might be associated with changes in cerebello-cerebral circuits *in vivo*.

Our experiments in GAD65-KO mice confirmed that the observed effect evoked by b78 administration is GAD65-specific. B78 induced a gradual and long-lasting depression in roughly 50% of the tested cells in rats and WT mice. By contrast, no depression was observed in any of the tested cells obtained from GAD65-KO mice. It is unclear why only 50% of Purkinje cells in cerebellar slices responded to treatment with b78 or b96.11. One possible explanation may be an unstable accessibility of GAD65Ab to intracellular GAD65. Internalization of antibodies by neurons, which enables the antibody to bind intracellular antigens, has been suggested (Geis et al., 2010; Manto et al., 2011), and was confirmed by us (Vega-Flores et al., 2014), but this process may depend on neuronal activities, or the internalized Ab concentration may be suboptimal. Alternatively, the residual presence of GABAergic vesicles in the nerve terminals may mask the effect of b78.

We investigated the mechanism by which blockade of GAD65 in the presynaptic terminals impaired GABA release. Besides generating GABA necessary for fine-tuning of GABA concentrations at the synapse, GAD65 is associated with the cytosolic face of GABAergic vesicles (Jin et al., 2003). Previous studies demonstrated that GAD65 mediates the shuttling of GABAergic vesicles to the synaptic cleft and that cleavage of GAD65 from the vesicles results in reduced GABA transport (Buddhala et al., 2012). Our co-immunoprecipitation results indicate that b78 and not b96.11 interferes with the binding of GAD65 to GABAergic synaptic vesicles. Our results however do not rule out that b78 acts also through its inhibition of GAD65 enzyme activity.

Based on our results, two independent events in the presynaptic mechanisms may cause the decrease in GABA release. First, b78 may reduce the release probability of GABAergic vesicles, as indicated by the decrease in the frequency of mIPSCs (Saitow et al., 2009). Second, b78 may inhibit GAD65 enzyme activity or block GAD65-mediated vesicular transport, as indicated by a tendency to smaller amplitudes of mIPSCS. These duplicated actions are in good agreement with previous reports that b78 inhibited the enzyme activity of GAD65 and interfered with the exocytosis of GABA-containing vesicle (Raju et al., 2005; Manto et al., 2007).

After having established that the *in vitro* application of b78 impaired the release of GABA, we addressed the b78-induced loss of cerebellar coordination *in vivo*. Therefore we conducted a series of experiments including classical eyeblink reflex, cerebro-cerebellar linkage, gait, and exploratory behavior.

The classical eyeblink conditioning test demonstrated that while blockage of GABAergic activity on cerebellar interpositus neurons by b78 did not modify learning curves, it resulted in significantly smaller conditioned responses during delay conditioning. These results suggest that b78 inhibits cerebellar circuits affecting the performance of conditioned eyelid responses, but not the acquisition process. Similar findings were reported in Lurcher mice (devoid of Purkinje cells; Porras-García et al., 2010), and reports in rabbits and cats indicate that alterations in the functional capabilities of cerebellar circuits produce significant changes in the performance of conditioned eyelid responses, but not in the learning curves (Welsh and Harvey, 1989; Jiménez-Díaz et al., 2004).

Our study of the effects of trains of HFS on the premotor area on corticomotor responses shows that infusion of b78 in the cerebellar interpositus nucleus of the rat markedly impairs the facilitatory effect exerted by the premotor cortex in a paired stimulation paradigm. This impairment was only observed for b78, suggesting that this effect is epitope-specific. Our results cannot be explained by a depressive effect of monoclonal GAD65Ab on spinal facilitation. Indeed, as we have shown previously, infusion of GAD65Ab in the interpositus nucleus enhanced the excitability of the spinal cord (Manto et al., 2007). In addition, we observed similar deficits using ISIs (between the medialis nerve and the contralateral M1) of up to 14 ms, excluding an impairment of spinal facilitation.

Finally, our behavioral findings show that administration of GAD65Ab to the cerebellum induced a significant decrease in exploratory behavior. Previously GAD65-KO mice were shown to exhibit increased anxiety-like responses (Kash et al., 1999). Abnormal GABAergic synaptic transmission is likely to result in gait and movement abnormalities, as suggested by the increased displacement times of b78-injected rats on the elevated beam. A recent study showed that the intrathecal passive transfer of IgG isolated from a SPS patient to rats induced anxiety-like behavior, with no obvious changes in activity levels in the open field (Hansen et al., 2013). The effect of the purified IgG on GAD65 enzyme activity was not investigated in this earlier study, therefore it is possible that differences in epitope specificities may result in these distinct behaviors.

**Figure 9:**
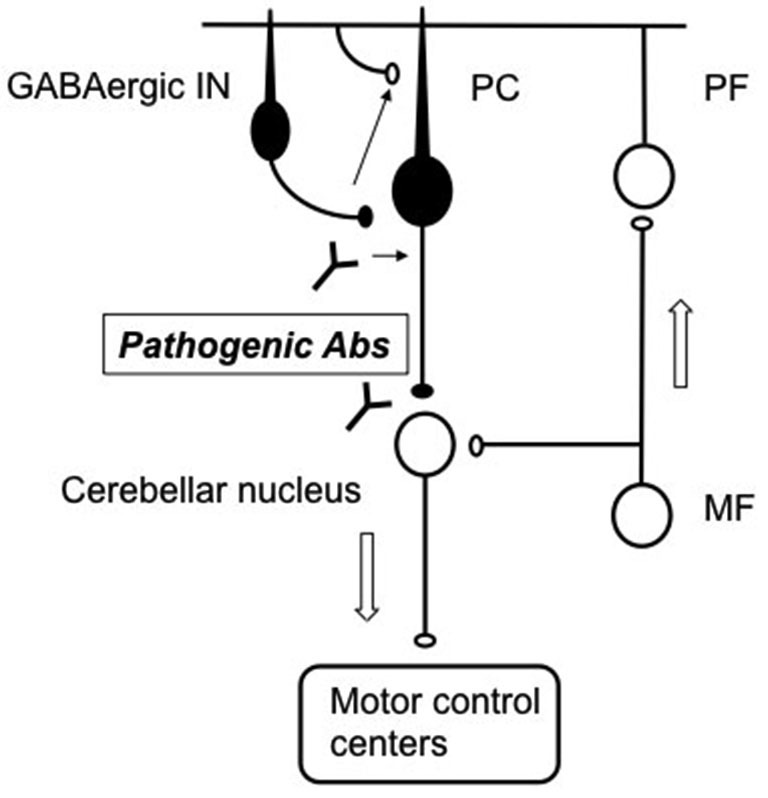
**Schematic diagram of pathogenic Abs in cerebellar circuits**. Pathogenic b78 acts on the terminals of GABAergic neurons to reduce GABA release. The decrease of GABA release alters the activities of PCs and cerebellar nuclei neurons, resulting in disorganized outputs to motor control centers. PC: Purkinje cells, GABAergic In: GABAergic interneurons, PF; parallel fibers, MF; mossy fibers. Arrows indicate flow of signals through the cerebellum. Excitatory neurons are indicated by white circles, while inhibitory neurons by black circles.

To our knowledge this is the first study that presents mechanistic detail on the pathogenic mechanism of monoclonal GAD65Ab on the cerebellum. The effects of monoclonal GAD65Ab mimicked those of patient’s IgGs in slices and in modulation on motor cortex *in vivo* (Ishida et al., 1999; Mitoma et al., 2003). Thus, our results support the idea that epitope specific GAD65Ab cause a disease-specific level of impairment in cerebellar circuits, leading to different neurological symptoms (Manto et al., 2011). We propose, that b78-mediated inhibition of GABA-release from GABAergic interneurons on cerebellar Purkinje cells elicits a dual impairment on synaptic transmissions on Purkinje cells; depression of inhibitory synapses and enhancement of excitatory synapses (Ishida et al., 1999; Mitoma et al., 2003; Figure 9). Furthermore, the dual impairment should occur similarly in cerebellar nuclei neurons. Consequently, the b78-induced deficits in GABA-release would elicit disorganized cerebellar outputs on various motor control centers. Unlike cerebral cortex pyramidal cells, Purkinje cells have no local inhibitory feed-back circuit that prevent burst activation and are thus maladapted to prevent hyperexcitability (De Schutter, 1995). Thus, the dual impairment, consisting of a decrease in GABA and an increase in glutamate, could cause the degeneration of Purkinje cells observed in ataxia.

Our results do not rule out a possible pathogenic role of other autoantibodies in ataxia and SPS. However the use of purified monoclonal GAD65-specific antibodies eliminates these factors from our analysis. Together with previous findings that the absorption of GAD65Ab completely diminished the CSF-induced depressive effect in rat cerebellar slices (Ishida et al., 2008), the present results support a pathogenic role of GAD65Ab. Our data suggest a possible therapeutic approach for the treatment of neurological patients with high GAD65Ab titers, via removal or selective blockage of b78-like GAD65Ab.

## Conflict of Interest Statement

The authors declare that the research was conducted in the absence of any commercial or financial relationships that could be construed as a potential conflict of interest.
